# Presentation of an Infant with Chromosome 18p Deletion Syndrome and Asymmetric Septal Hypertrophy

**DOI:** 10.1055/s-0042-1743261

**Published:** 2022-02-25

**Authors:** Ayca Kocaaga, Sevgi Yimenicioglu

**Affiliations:** 1 Department of Medical Genetics, Health Ministry Eskisehir City Hospital, Eskişehir, Turkey; 2 Department of Child Neurology, Health Ministry Eskisehir City Hospital, Eskişehir, Turkey

**Keywords:** asymmetric septal hypertrophy, 18p deletion syndrome, dysmorphic features, intellectual disability, chromosomal microarray analysis

## Abstract

The frequency of 18p deletion syndrome is estimated to be ∼1/50,000 live births and is more commonly associated with certain clinical features including short stature, intellectual disability, and facial dysmorphism. Physical examination of our patient revealed a short stature, intellectual disability, facial dysmorphism (microcephaly, ptosis, epicanthus, low nasal bridge, protruding ears, long philtrum, and thin lips), and clinodactyly of the fifth finger. The peripheral karyotype was 46, XX, del (18) (p11.32p11.2). DNA microarray analysis revealed a de novo 13.9-Mb deletion at 18p11.32p.11.21. Echocardiography revealed asymmetric septal hypertrophy. Congenital cardiac abnormalities are present very rarely in this syndrome. This finding suggests that one locus or loci that play a role in cardiac development is located in this chromosomal region. Although rare, cardiac hypertrophies should be kept in mind when evaluating a patient with phenotypic anomalies and genetic results compatible with an 18p deletion syndrome.

## Introduction

The 18p deletion syndrome (MIM 146390) is a rare chromosomal disorder characterized by deletion of the short arm of chromosome 18. 1 The prevalence is estimated to be ∼1 in 50,000 live births. 2 It was first recognized in 1963 by Jean de Grouchy, with ∼150 cases reported to date. 3 Clinical features vary considerably between patients. The clinical abnormalities include short stature, intellectual disability, facial dysmorphism (microcephaly, ptosis, epicanthus, low nasal bridge, protruding ears, long philtrum, and thin lips), and clinodactyly of the fifth finger. 4 However, skeletal or brain malformations, mostly of the holoprosencephaly, have also been reported. 5 6 7 In most patients (>75%), deletions are seen de novo. 8 The rest are caused by de novo translocation or imbalanced familial transmission of structural rearrangements. 6 Clinical manifestations tend to be related to the size of the deletion and the genes affected. In this syndrome, the phenotype–genotype correlation is still not established definitively. 9 Here, we report a male infant with del(18p) with asymmetric septal hypertrophy and also review the literature

## Case Report

**Fig. 1:**
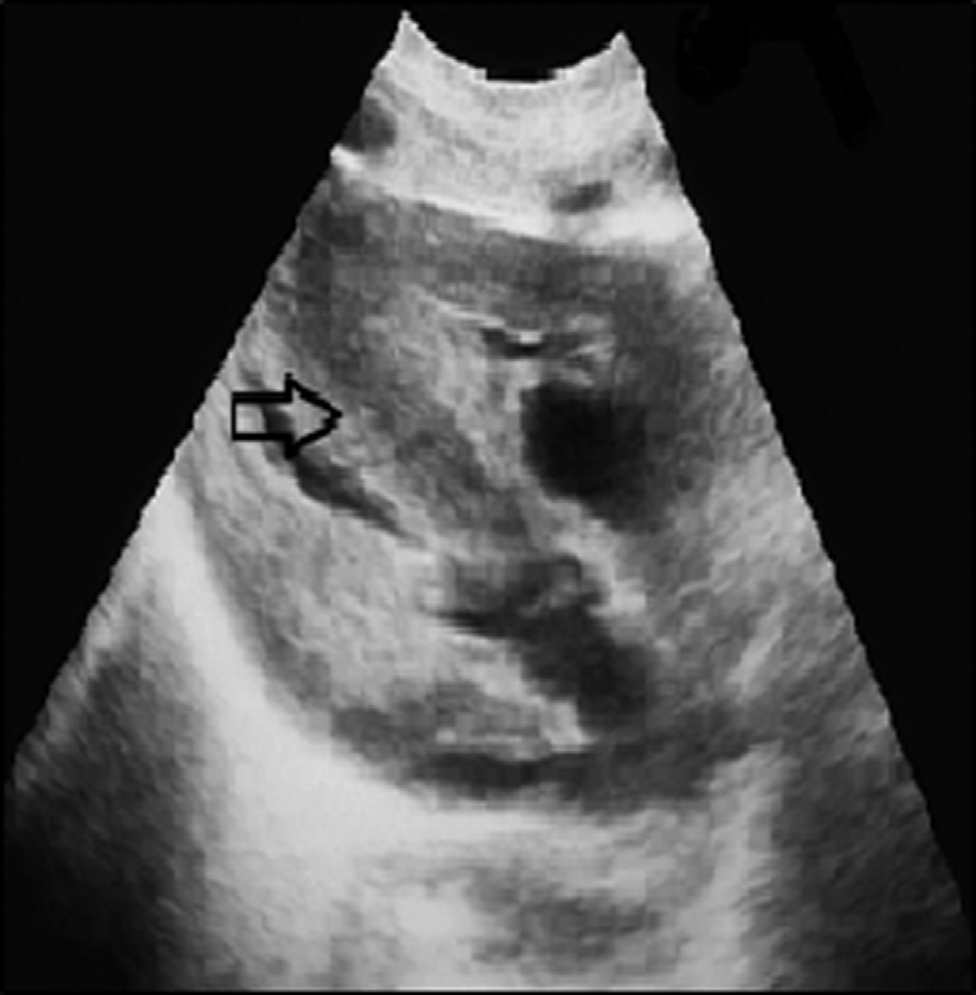
Echocardiography demonstrating asymmetrical hypertrophy of the interventricular septum. The arrow is an indication of the echocardiography of the patient showing asymmetric septal hypertrophy.

**Fig. 2:**
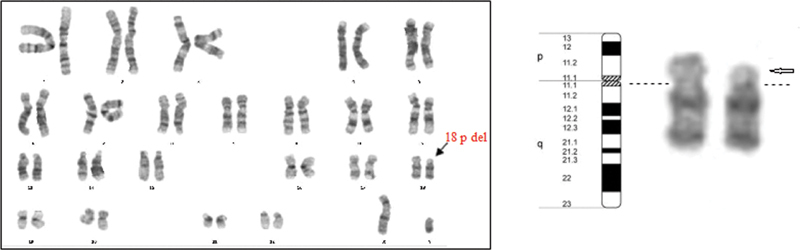
The karyotype showing deletion of the short arm of chromosome 18. Chromosome analysis at 550-band resolution revealed 46,XY,del(18)(p11.32p.11.21).

**Fig. 3:**
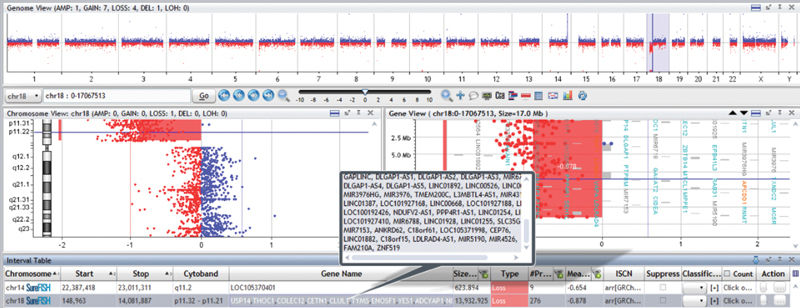
Array comparative genomic hybridization (CGH) results showing 18p deletion (148,963–14,081,887).

He was born with a weight of 2,720 g (10th–25th percentiles) and height of 46 cm (3rd–10th) by normal vaginal delivery at 37 weeks of gestational age without perinatal problems. He was the second child of healthy nonconsanguineous Turkish parents. There was no family history of hereditary disease or mental retardation. The patient showed a long face, down-slanted palpebral fissures, broad nasal bridge, long philtrum, thin lips, high-arched palate, malocclusion, and protruding ears. Developmental milestones were retarded; he was able to sit at 9 months and walk at 21 months, but cannot speak yet. His ophthalmological and audiological examinations were normal. Cerebral imaging and abdominopelvic ultrasound were performed, which revealed no abnormalities. He was operated on for bilateral cryptorchidism. This was followed by child cardiology due to asymmetric septal hypertrophy ( Fig. 1 ). Chromosomal analysis from peripheral blood lymphocytes revealed an unknown deletion of the short arm of chromosome 18 [46,XX, der(18)t(18;?)(p11.1;?)] ( Fig. 2 ). The proband's mother revealed a 46-XX karyotype and his father a 46-XY karyotype. Agilent oligonucleotide microarray to investigate for copy number variants was done using the 8 × 60K probe. The CMA revealed a 13.9-Mb deletion in 18p11.32p.11.21, defined as arr18p11.32p11.21[148,963–14,081,887]x1 ( Fig. 3 ). The microarray analysis of the parents did not have a variation in copy number. These results showed that the deletion of chromosome 18 in the patient was de novo. The consent form for the publication of this report and the accompanying images has been signed by the parents of the child.

## Discussion

The 18p deletion syndrome is a rare chromosomal disorder resulting from a partial deletion of the short arm of chromosome 18. 10 At least 150 cases of 18p deletion syndrome have been reported. 2 The common clinical features include minor facial anomalies, neurodevelopmental problems, genitourinary abnormalities, hypotonia, obesity, and epilepsy. 10 Dysmorphic facial features, neurodevelopmental delay, short stature, cryptorchidism, and asymmetrical septal hypertrophy were observed in our patient. The patient reported here has a severe form of 18p deletion syndrome, presenting most of the characteristic features of this condition.

Grosso et al reported that 18p deletion was less likely to be associated with epilepsy. However, the patient had no history of epilepsy. 11 The short stature seen in our patient is another feature of this syndrome. There are some reports suggesting that growth hormone (GH) deficiency occurs with deletions of the 18p distal region. 12 13 The growth delay of our patient may be the result of the deletion of this region.

The cardiac abnormalities in cases with 18p deletion syndrome are reported very rarely. Digilio et al reported a left atrial isomerism in a patient with 18p deletion. 14 Wester et al reported an atrial septal defect, ventricular septal defect, and tricuspid atresia in three patients with 18p deletion syndromes in 2006. 5 Cardiac examination of our patient revealed asymmetric septal hypertrophy. This finding has not yet been reported in the literature. There are more than 12 genes related to this syndrome, contributing to the variable clinical pictures. 15 It is not known exactly the complete deletion of which of these genes causes hypertrophy in the heart. However, when the literature is examined in detail, it has been reported that deletions containing the *NDUFV2* gene in this region cause early-onset hypertrophic cardiomyopathy and encephalopathy. Bénit et al identified a homozygous 4-bp deletion in the *NDUFV2* gene in an infant with early-onset hypertrophic cardiomyopathy and encephalopathy. 16 Pagniez-Mammeri et al reported the same mutation (4-bp deletion, IVS2 + 5_ + 8delGTAA in the *NDUFV2* gene) in a patient with hypertrophic cardiomyopathy and encephalopathy. 17 Cameron et al showed a compound heterozygosity (c.IVS2 + 1delGTAA and c.669_670insG) in the *NDUFV2* gene in a patient with hypertrophic cardiomyopathy. 18 18p deletions containing *NDUFV2* appear to be associated with asymmetric septal hypertrophy as in our patient.

## Conclusion

In conclusion, we detected an important phenotypic variability in cardiac abnormalities in patients with 18p deletion syndrome. Although cardiac anomalies are reported in ∼5 to 10% of patients with 18p deletion, asymmetric septal hypertrophy has never been reported before. Deletion of the *NDUFV2* gene is probably responsible for this phenotype in patients with 18p deletion syndrome. Further studies with extended patient cohorts and detailed genomic mapping of the 18p region are needed to elucidate the genotype–phenotype correlations in this rare syndrome. Therefore, more studies are needed to determine the etiology and the relationship between genes and phenotypes.
